# The Critical Role of Biliary Candidiasis in Development of Surgical Site Infections after Pancreatoduodenectomy: Results of Prospective Study Using a Selective Culture Medium for Candida Species

**DOI:** 10.1155/2018/5939724

**Published:** 2018-11-18

**Authors:** Hiroyuki Kato, Yusuke Iizawa, Kei Nakamura, Kazuyuki Gyoten, Aoi Hayasaki, Takehiro Fujii, Yasuhiro Murata, Akihiro Tanemura, Naohisa Kuriyama, Yoshinori Azumi, Masashi Kishiwada, Shugo Mizuo, Masanobu Usui, Hiroyuki Sakurai, Shuji Isaji

**Affiliations:** Department of Hepatobiliary Pancreatic and Transplant Surgery, Mie University Graduate School of Medicine, Japan

## Abstract

In accordance with previous reports, the incidence of biliary candidiasis (BC) after pancreaticoduodenectomy (PD) was reported to be 0 to 5%, and the clinical significance of BC still has been elusive. In this study, we prospectively evaluated the precise incidence of BC after PD using the CHROMagar Candida plate in an attempt to elucidate whether BC has a significant impact on the clinical outcomes after PD.* Patients and Method*. From November 2014 to March 2016, the consecutive 51 patients who underwent PD were enrolled for this study. The bile juice was prospectively collected through the biliary stent tube on postoperative days (POD) 3, 7, and 14 and directly incubated onto the CHROMagar Candida plate for the cultivation of various Candida species. In the presence or absence of BC, we compared the incidence of SSIs.* Results*. The incidence of postoperative BC was 15% on POD 3, 24% on POD 7, and 39% on POD 14, respectively. Taken together, 22 patients out of 51 (43.1%) developed BC after PD. Moreover, the incidence of SSIs was significantly higher in patients with BC than in those without it (71% versus 7%, p=0.005). BC was selected as the only significant risk factor of SSIs after PD among the various risk factors. Even though a cause of BC is unknown, high level of alkaline phosphatase (cut-off line >300 IU/L) was selected as the only preoperative risk factor of the development of BC.* Conclusion*. We elucidated new evidence in which BC could be the independent cause of SSIs after PD and should not be recognized as just contamination artifacts. Preoperative assessment for identifying carriers of Candida species might be essential for reducing the incidence of SSIs after PD.

## 1. Introduction

Candida species play a critical role in nosocomial, catheter, and skin infections in recent years, especially in patients with malignancies, long-term antibiotic therapy, diabetes mellitus with multiorgan complications, intensive care unit (ICU) admission, immunosuppressive drugs, and posttransplant status [1–4]. Even though the importance of Candida infections has been increasing in recent years, impact of biliary candidiasis (BC) on complications after pancreaticoduodenectomy (PD) and its incidence are still unknown. On the other hand, BC is generally diagnosed when microbiological bile fluid analysis detects candida species, whereas the pathogenicity of its presence might be regarded as an unrecognized clinical problem unless patients have a significant symptom [5].

Recently, our retrospective study newly revealed that the incidence of patients with BC frequently developed severe infectious complications including abscess formation compared with patients without BC, and Candida species in bile juice was identified as the independent risk factors of SSIs after PD [6]. These results made us reconsider a significance of BC after PD even if it seems to be an inapparent infection. However, our previous study has potential limitations because the study retrospectively assessed the patients who underwent PD, resulting in high selection bias and made us conduct the further prospective study.

CHROMagar Candida plates include a chloramphenicol for inhibiting a growth of other bacterial species and allow us to instantly differentiate various Candida species just by the color of the colony. In fact, several studies revealed that the detection rates for polymicrobial species using CHROMagar Candida agar plates were 20% higher than that using the Sabouraud-chloramphenicol agar plates, which is also previous traditional media for detecting yeast species [7]. In our institution, this plate has been routinely used for detecting yeast species with concurrent bacterial culture, resulting in 37.5% positive rates of Candida species in bile juice after PD and allowed us to show that the incidence of BC after PD was markedly high compared to the previous reports [8, 9].

In this study, we prospectively conducted the microorganism culture of bile juice on postoperative days (POD) 3, 7, and 14 using CHROMagar, leading to obtaining the precise incidence of BC after PD. Moreover, according to the presence or absence of positive candida cultures, we compared the incidence of SSIs and evaluate the perioperative risk factor of BC. The aim of this study is to elucidate a new insight in which BC could be the independent cause of SSIs after PD and should not be recognized as just contamination artifacts.

## 2. Patients and Methods

### 2.1. Patients

From November 2014 to March 2016, the consecutive 51 patients who underwent PD were enrolled for this study. This study was approved by the Ethical Committee of Mie University Hospital (No. 2927) and informed consent was obtained from all patients. According to our previous retrospective study, clinical significance of BC could be obtained by 56 cases number. Therefore, we planned to set the sample size at 50 to 60 patients who underwent PD.

### 2.2. Surgical Procedures and Perioperative Administrations

Preoperative biliary drainage was performed by endoscopic retrograde biliary drainage if obstructive jaundice was seen. Probiotics treatments such as Lactobacillus products were conducted preoperatively at the outpatient clinic if necessary.

In terms of the surgical procedures, our institution has usually employed subtotal stomach-preserving PD regardless of the type of disease. After cutting a hepatic duct, 8 to 16 Fr Nelaton catheter was inserted according to the size of stump, and bile juice was externally drained until hepaticojejunostomy for avoiding intraoperative bile spillage.

Reconstruction was carried out by a modified Child method; end-to-side pancreaticojejunostomy, end-to-side hepaticojejunostomy, and end-to-side gastrojejunostomy. In pancreaticojejunostomy, we conducted a pair-watch suturing technic, allowing us to standardize the anastomosis regardless of the pancreatic texture and the diameter of main pancreatic duct (MPD) [10]. The 5Fr pancreatic stent tube was usually placed in the cases with soft pancreas, narrow MPD, and/or severe comorbidity. As of the hepaticojejunostomy, instead, we routinely placed the 5Fr biliary stent tube, which was guided externally through the jejunal loops. Before skin closure, prophylactic wound irrigation was conducted for all 51 patients for avoiding SSI. A feeding jejunostomy tube was placed intraoperatively for early postoperative enteral nutrition. Just after the surgery, Flomoxef sodium was used as the prophylactic antimicrobial therapy and it was continued until POD 3 at least. A 19F closed suction drain was placed in Winslow's foramen and removed until POD 5 as long as drain discharge was clear and its amount was less than 200ml/day.

### 2.3. Postoperative Biliary Cultures and Diagnosis of Biliary Candidiasis

On postoperative days 3, 7, and 14, bile juice collected through the biliary stent tube was directly incubated onto CHROMagar Candida plate for the cultivation of various fungal species, especially Candida species, and appropriate agar plates were also prepared concurrently for the cultivation of bacterial species. The microorganism cultures were performed unless the biliary stent tube was dislocated into the jejunum or clamped. Intraoperative bile juice culture was not included in this prospective study, because Candida species was rarely detected in intraoperative bile juice based on our experiences.

The bile juice collected through the biliary stent tube was directly incubated onto CHROMagar Candida plate for the cultivation of various Candida species. CHROMagar Candida plate was used according to the instructions given by the manufacturer (Kanto Chemical Co., Inc., Tokyo, Japan). The growth was checked every 24 hours until 72 hours of incubation and determination of species was carried out according to the colors of colonies:* C. albicans *in green,* C. glabrata* in purple,* C. tropicalis* in blue surrounded by a pink halo, and* C. parapsilosis* and* C. krusei* in pink with downy appearance. For the cultivation of bacterial species, a smear of gram stain was examined to select the appropriate agar gel. When gram-positive bacteria were found in a smear of gram stain, m-Enterococcus agar (BD Biosciences, Tokyo, Japan) or CHROMagar MRSA/Staph plates (Kanto Chemical Co., Inc., Tokyo, Japan) were used for detecting bacterial species. When gram-negative bacteria were seen in a smear, ChromID CPS agar (BioMérieux, Tokyo, Japan) or MacConkey II agar plates (BD Biosciences, Tokyo, Japan) were chosen for detecting bacterial species. On the other hand, any bacteria could not be seen in a smear of gram stain, we chose the blood agar plate (Kanto Chemical Co., Inc., Tokyo, Japan) or MacConkey II agar (BD Biosciences, Tokyo, Japan) as the nonselective bacterial plates. The growth was checked every 24 hours until 120 hours of incubation at 35°C. Isolates grown from nonselected medium were identified with a bacteriologic automated system (VITEC System™, BioMérieux, Tokyo, Japan)

### 2.4. Evaluation of Risk Factors of BC

We selected the variables from pre- and intraoperative factors that may influence the development of BC. Preoperative factors included age, sex, body mass index (BMI), diabetes mellitus, preoperative biliary stenting, nutritional status including levels of total protein, albumin, and total cholesterol, NLR (neutrophil lymphocytes ratio), and primary disease (malignant tumor or not). Intraoperative factors included operation time, amount of blood loss, resections of portal vein (PV), hepatic artery, and other organs.

### 2.5. Surgical Site Infections

According to the Centers for Disease Control and Prevention (CDC) guideline, SSIs were categorized as incisional SSIs, deep incisional SSIs, and organ or space infection which involves any part of the internal organs other than the incision that is treated with the surgical procedure or interventional approach. Moreover, in accordance with Fujii T et al., incisional SSIs could be classified into five categories as follows: Grade 0, no sign of infection; Grade 1, only redness or swelling of the wound, not requiring any treatment; Grade 2, an infection involving only the skin or subcutaneous tissue; Grade 3, infection involving the fascial and muscle layers; and Grade 4, infection invading the organ/space that drains through the incision [11]. In this study, we defined the patients with SSIs when the incisional SSIs greater than Grade 2 and/or organ or space SSIs were found from the date of surgery to discharge.

Median follow-up days were 35.

### 2.6. Statistical Analysis

The results of the continuous variables were expressed as mean values with SD and the statistical significance was determined by Student's t-test. Dichotomous variables were evaluated by chi-square analysis or Fisher's test, as appropriate. Only variables whose p value was less than 0.1 by univariate analysis were included in the multivariate analysis, which was performed using a multiregression analysis. Results were considered significant when the P values were less than 0.05. To assess the optimal cut-off values of risk factors for BC, receiver-operating characteristic (ROC) curves were used.

## 3. Results

In terms of the backgrounds of all 51 patients, as shown in Table 1, mean age was 66.6±9.3 years, and male and female were 29 and 22, respectively. Histological diagnosis was pancreatic carcinoma in 29, intraductal papillary-mucinous neoplasm (IPMN) in 9, bile duct carcinoma in 7, carcinoma of ampulla in 2, and others in 4. In nutritional status, BMI, total protein, and albumin level were 20.3±3.7 kg/m2, 6.5±0.73 g/dl, and 3.9±0.45 g/dl, respectively. The 8 patients (15.7%) were diagnosed as diabetes mellitus based on previous history or laboratory data. Preoperative biliary drainages were performed in 16 (33.3%). Preoperative cholangitis greater than Grade 1 based on Tokyo guideline 13 [12] developed in 11 (21.6%). Preoperative chemoradiotherapy was conducted in 26 (51.0%).

On POD 3 and 14, bile juice cultures could not be performed in 3 and 15 patients, respectively, since we could not collect sufficient amounts of bile juice, or stent tubes were dislocated or clamped for ready to discharge. When we aggregated the results of bile cultures on POD 3, 7, and 14, Enterococcus species were most frequently found in 37 patients (72.5%), followed by Candida species in 22 (43.1%) and Enterobacter species in 12 (23.5%). Therefore, the incidence of postoperative BC was comparable to 37.5% in our previous retrospective study [6]. In detail, C. albicans were found in 18,* C. glabrata* in 6, and other species in 1 (Table 2). The detection rate of Candida species according to POD were 15% (7/48) on POD 3, 24% (12/51) on POD 7, and 39% (14/36) on POD 14.

The incidence of SSIs was 27.5% (14/51) in this study and incisional and organ/space SSIs were found in 5 and 9, respectively. As of the postoperative complications related to anastomotic leakage, clinically relevant pancreatic and biliary fistula were seen in 1 (2.0%) and 2 (3.9%), respectively. We compared the incidences of SSIs and early postoperative complications between the 22 patients with BC and 29 without BC. The incidence of postoperative SSIs after PD was significantly higher in the cases with BC than that without it: 71% versus 6.8% (p=0.005). Moreover, univariate analysis identified that BC was the only risk factor of SSIs after PD and any risk factors such as BMI, operation time, blood loss, history of cholangitis, and preoperative biliary drainage were not selected as the risk factors of SSIs. On the other hand, BC and blood transfusion were selected as the independent risk factor of SSI after PD by multivariate analysis (Table 3). Moreover, Candida species were only significant risk factor of SSIs and were highly detected (60%, 6/10) in the cultures from the sites of infections especially when BC were found. When we focused on the specific species of Candida, the incidence of SSIs was significantly higher in patients with* C. glabrata* than in those with other Candida species 100% (6/6) versus 37.5% (6/16) (p=0.032).

To identify the risk factors of BC after PD, we compared pre-, intra-, and early postoperative characteristics between the patients with and without BC. By univariate analysis, preoperative usage of antibiotics, higher levels of total protein, albumin, and alkaline phosphatase, no combined portal vein resection, and higher PT-INR POD 1 were selected as significant risk factors for BC (Table 4). Multivariate analysis revealed that preoperative alkaline phosphatase and PT-INR on POD 1 were selected as the significant independent factors for BC after PD (Table 4). ROC curves revealed that optimal cut-off values of ALP and PT-INR to determine whether BC occurs or not postoperatively were 297U/l and 1.115, respectively.

## 4. Discussion

Our prospective study newly revealed the following 3 things: (1) the incidence of BC after PD was 43.1% when Candida specific plate was used, and it was considered to be higher incidence compared to previous reports [8, 9]. (2) BC potentiates the incidence of SSIs after PD and was selected as the most influential risk factor of SSIs. (3) The precise etiology of BC was still unknown, but preoperative high ALP levels and prolonged PT-INR on POD 1 were significantly related to the development of BC.

Several reports have found that preoperative biliary drainage increases the rate of postoperative infectious morbidity including SSIs after PD [13–15]. Sudo et al. reported that prophylactic antibiotics effectively targeted approximately 30% of bile microorganisms in patients with preoperative biliary drainage and therefore recommended the perioperative usage of antibiotics according to the result of preoperative biliary culture [8]. However, these papers were focusing on only bacterial species, and to the best of our knowledge, there had been no article focusing on BC and SSIs after PD until our first previous retrospective study pointed out its significance in 2016 [16]. A potential limitation of our previous study is that we retrospectively assessed 56 patients who underwent PD, resulting in high selection bias.

Our present prospective study showed that BC was selected as the most significant risk factor of SSIs after PD, and unexpectedly history of cholangitis, BMI, and long operation time were not selected as risk factors. However, its underlining mechanism that BC increased SSIs has yet to be addressed. According to the previous experimental studies on fungal-bacterial interactions using a mice model, Carson et al. have concluded that Candida infection process not only causes physical damage to organ walls, allowing other microorganisms to penetrate more easily, but also directly stimulates the growths of* Staphylococcus aureus, Serratia marcescens, and Streptococcus faecalis* [17]. This fungal-bacterial coinfection ability to damage tissue would explain the reason why BC increases SSIs. More recent studies on fungal-bacterial interaction, however, have revealed that interaction between Candida species and most of bacterial species has synergetic effect, but that the presence of pseudomonas species invariably inhibits Candida species growth, showing antagonistic effect [18, 19]. Therefore, precise details of the unique biology of fungal-bacterial interaction should be more extensively explored in the future. Based on these articles, efforts to avoid intraoperative bile spillage should be absolutely important because we believe that bile spillage, especially spillage of Candida species, into the abdominal also causes both organ/space and incisional SSIs [20, 21]. Even though most of the BC has been regarded as asymptomatic candidiasis or contamination artifact, our prospective data could validate the prophylactic antifungal therapy for BC, when patients are exposed to prolonged bile spillage during surgery.

The precise etiology of BC is still unknown. According to the recent retrospective and prospective studies for BC among the patients who underwent ERCP, long-term antibiotic therapy, history of immunosuppressive treatment, history of endoscopic biliary sphincterotomy, and old age were identified as the significant risk factors of BC [5, 22]. Therefore, we hypothesized that these variables could be identified as influential risk factors causing BC after PD. However, the presence of preoperative biliary stenting, preoperative cholangitis and preoperative chemoradiotherapies, neutrophil lymphocyte ratio (NLR), nutrition status, intraoperative blood loss, and operation time were excluded as the risk factors of BC by even univariate analysis. Unexpectedly, preoperative high ALP levels and prolonged PT-INR on POD 1 were significantly related to the development of BC. These results seem to be difficult to interpret. Candida species have been recognized as one of the main causes for acute acalculous cholecystitis, which are usually induced by bile stasis in cases of long fasting period [23, 24]. Moreover, the previous literature reported that routine bile collection for microbiological analysis during cholangiography revealed that Candida species were detected in 68 (10%) cases out of 689 detected microorganisms [25]. Therefore, we considered that several patients already had Candida species in bile juice as commercial microorganism, and these Candida species latently potentiate intrabiliary pressure, which might be leading to potential bile stasis, elevation of ALP, and prolonged PT-INR. Based on these data, the patients with high level of ALP, especially more than 300 U/L preoperatively, could be recognized as the potential careers of Candida species. However, further study should be conducted to confirm our hypothesis.

ALP and PT-INR are considered to be insufficient to fully explain the cause of BC. Candida species are recognized as a commensal organism in the otopharyngeal cavity and gastrointestinal tract [26], and especially,* C. albicans* can be detected as normal large-bowel flora in about 50% of individuals [27]. Lenz et al. revealed the high concordance between Candida species detected in the gut and bile juice and underlined the possibility of an infectious pathway from the intestine [22]. Therefore, we considered that preoperative insidious Candida infection within GI tract could cause the ascending Candida infection through the hepaticojejunostomy. To explore the incidence of commensal Candida infections in gastrointestinal tract, we evaluated the preoperative microorganism cultures of gastric juice among 28 patients in whom PD was proposed, and* C. albicans and C. glabrada *in gastric juice were detected in 10 and 3 patients, respectively, and these fungal species were cultured from the postoperative bile juice in 6 of 10 (60%) and 2 of 3 (67%), suggesting that preoperative Candida infection might cause the ascending infection through the hepaticojejunostomy (data not shown). Therefore, preoperative exploration of Candida species might be informative and clinically relevant for selecting potential careers of Candida species.

When we focused on the treatment for BC, preoperative oral amphotericin B, which is unabsorbable from guts, might be effective for reducing the incidence of BC and SSIs after PD, especially among Candida careers because it effectively reduces the number of fungal flora in guts without adverse effect. Further study should be needed to show whether or not prophylactic antifungal therapy is effective for reducing SSIs. The limitation of this study is its single-institutional review, which brings a several bias such as the diversity of surgical procedures perioperative patient administrations. This limitation will need to be verified with multi-institutional reviews and possible clinical studies.

In conclusion, biliary candidiasis (BC) significantly increased the incidence of SSIs after PD. Even though a cause of BC is unknown, high level of alkaline phosphatase (cut-off line >300 IU/L) was selected as the only preoperative risk factor of BC. The exploration of potential career of Candida species might be necessary for predicting the occurrence of postoperative BC and SSIs after PD.

## Figures and Tables

**Table 1:** The comparison of preoperative backgrounds between our prospective and retrospective studies.

Patient's background	n=51
Age (mean±SD)	66.6±9.3
Gender (male/female)	29/22
Preoperative diagnosis PDAC/IPMN/BDC/AC/others	29/9/7/2/4
Body mass index (kg/m2)	20.3±3.7
Preoperative total protein (g/dl)	6.5±0.73
Preoperative albumin (g/dl)	3.9±0.45
Preoperative cholinesterase	0.79±0.20
Preoperative diabetes mellitus (yes/no)	8/43
Preoperative biliary drainages (yes/no)	16/35
Preoperative cholangitis (yes/no)	11/40
Preoperative usage of broad antibiotics (yes/no)	29/22
Preoperative chemoradiotherapy (yes/no)	26/25

PDAC: pancreatic ductal adenocarcinoma, IPMN: intraductal mucinous neoplasm, BDC: bile duct carcinoma, and AC: ampullary carcinoma.

**Table 2:** Detail of bacterial and fungal species from biliary cultures.

Bacterial and fungal species	n
**Enterococcus species**	37
Unknown or other species	19
*Enterococcus faecalis*	14
*Enterococcus faecium*	6
**Enterobacter species**	12
**Klebsiella species**	9
**Staphylococcus species**	7
*Pseudomonas aeruginosa*	5
*Escherichia coli*	4
Candida species	22
*Candida albicans *	18
*Candida glabrata*	6
Unknown Candida species	1

**Table 3:** Uni- and multivariate analysis for identifying the risk factors of SSIs after PD.

Perioperative variables	SSIs (n=14)	Non-SSIs (n=37)	P-value	**Odd's ratio**	**95**%** CI**	**P-value**
**Preoperative factors**						
Age (mean±SD)	69.6±6.8	65.5±10.0	0.220			
Gender (male/female)	10/4	19/18	0.196			
Primary disease (malignancy/benign)	11/3	28/9	1.00			
Preoperative biliary stenting	6/8	11/26	0.508			
Preoperative Chemoradiotherapy (yes/no)	6/8	20/17	0.475			
Preoperative usage of antibiotics (yes/no)	9/5	20/17	0.510			
Preoperative probiotics usage (yes/no)	2/12	12/25	0.297			
Preoperative diabetes mellitus	1/13	7/30	0.491			
Body mass index (kg/m2)	20.6±3.9	20.2±4.0	0.874			
Total protein (g/dl)	6.9±0.79	6.5±0.69	0.080			
Albumin (g/dl)	4.0±0.42	3.9±0.47	0.429			
Cholinesterase	0.79±0.19	0.79±0.21	0.958			
White blood cell counts (/mm2)	5,393±1,801	5,252±1,568	0.908			
Neutrophils/lymphocytes ratio	3.6±1.8	3.4±2.5	0.353			
Hemoglobin (g/dl)	12.5±1.6	12.2±1.3	0.526			
Platelet count (x10^3^ ml)	213±79	207±49	0.393			
Total bilirubin	0.70±0.36	0.70±0.35	0.348			
Direct bilirubin	0.22±0.17	0.15±0.11	0.128			
Aspartate aminotransferase (U/l)	24±10.5	23±17	0.305			
Alanine aminotransferase (U/L)	25±15	19±16	0.266			
C-reactive protein (mg/l)	0.35±0.15	0.20±0.25	0.434			
Lactic acid dehydrogenase (U/l)	176±58	189±36	0.966			
Gamma-glutamyl transferase (U/l)	98±127	32±40	0.148			
Alkaline phosphatase (U/l)	301±141	263±107	0.291			
**Bacterial and fungal species through bile juice**						
Enterococcus species (yes/no)	13/1	24/13	0.077			
Klebsiella species (yes/no)	2/12	7/30	0.981			
*Escherichia coli *(yes/no)	1/13	3/34	0.639			
*Pseudomonas aeruginosa *(yes/no)	2/12	3/34	0.606			
Enterobacter species (yes/no)	3/11	9/28	0.879			
Staphylococcus species (yes/no)	2/12	5/32	0.701			
Candida species (yes/no)	12/2	10/27	**<0.001**	**333**	**0.003-0.28**	**0.0002**
**Intra- and early postoperative factors**						
Operation time (min)	490±165	499±114	0.696			
Other organ resections (yes/no)	1/13	1/36	0.478			
Combined portal vein resections (yes/no)	3/11	19/18	0.054			
Intraoperative blood loss (g)	730.5 (216-7700)	491 (0-4930)	0.696			
Blood transfusion (yes/no)	6/8	6/31	0.066	**11.5**	**0.009-0.832**	**0.034**
PT-INR (POD1)	1.29±0.14	1.21±0.15	0.105			
C-reactive protein on POD1(mg/l)	8.6±2.9	7.6±3.1	0.221			
White blood cell count POD1	9,746±5,292	9,319±3,182	0.736			

POD: postoperative day and PT-INR: prothrombin time-international normalized ratio.

**Table 4:** Uni- and multivariate analysis for identifying the risk factors of BC followed by PD.

**Perioperative variables**	**BC (n=22)**	**Non BC (n=29)**	**P-value**	**Odd's ratio**	**95**%** CI**	**P-value**
**Preoperative factors**						
Age	64.9±10.9	67.9±8.0	0.440			
Gender (male/female)	14/8	15/14	0.196			
Primary disease (malignancy/benign)	15/7	24/5	0.224			
Preoperative diabetes mellitus (yes/no)	4/18	4/25	0.713			
Preoperative biliary stenting (yes/no)	8/14	9/20	0.689			
Preoperative cholangitis (yes/no)	6/16	5/24	0.498			
Preoperative CRT (yes/no)	9/13	17/12	0.210			
**Preoperative history of antibiotics usage (yes/no)**	**16/6**	**13/16**	**0.046** **∗**			
Preoperative probiotics usage (yes/no)	4/18	10/19	0.196			
BMI (kg/m2)	19.9±3.7	20.7±3.9	0.435			
**Preoperative factors**						
**Total protein (g/dl)**	**6.9±0.67**	**6.4±0.69**	**0.011** **∗**			
**Albumin (g/dl)**	**4.1±0.45**	**3.7±0.41**	**0.012** **∗**			
Choline esterase	0.83±0.22	0.75±0.19	0.196			
White blood cell counts (/mm2)	5,441±1,272	5,176±1,851	0.171			
Neutrophils/lymphocytes ratio	3.3±1.5	3.6±1.8	0.704			
Hemoglobin (g/dl)	12.8±1.6	11.9±1.2	0.045			
Platelet count (x10^3^/ml)	235±79	197±49	0.092			
Total bilirubin	0.76±0.35	0.69±0.35	0.442			
Direct bilirubin	0.17±0.14	0.17±0.13	0.831			
Aspartate aminotransferase (U/l)	22±9.4	24±18	0.429			
Alanine aminotransferase (U/L)	22±13	20±18	0.244			
C-reactive protein (mg/l)	0.25±0.27	0.25±0.39	0.689			
Lactic acid dehydrogenase (U/l)	188±53	183±34	0.213			
Gamma-glutamyl transferase (U/l)	55±51	88±58	0.842			
**Alkaline phosphatase (U/l)**	**327±134**	**232±63**	**0.004** **∗**	**1.103**	**1.001-1.019**	**0.027**
**Intra and early postoperative factors**						
Operation time (min)	497±148	495±114	0.621			
Other organ resections (yes/no)	1/21	1/28	1.00			
**Combined portal vein resections (yes/no)**	**6/16**	**16/13**	**0.046** **∗**			
Intraoperative blood loss (g)	646 (0-7700)	497 (75-4930)	0.932			
Blood transfusion (yes/no)	5/17	7/22	0.257			
**PT-INR (POD1)**	**1.30±0.14**	**1.19±0.15**	**0.018** **∗**	**267.4**	**1.173-60,932**	**0.044**
C-reactive protein on POD1(mg/l)	8.0±3.3	7.7±2.8	1.00			
White blood cell count POD1	9,445±3,959	9,421±3,647	0.983			

POD: postoperative day, PT-INR: prothrombin time-international normalized ratio.

## Data Availability

All data generated or analyzed during this study are included within the article.
